# Effects of second-generation antipsychotics on selected markers of one-carbon metabolism and metabolic syndrome components in first-episode schizophrenia patients

**DOI:** 10.1007/s00228-014-1762-2

**Published:** 2014-10-08

**Authors:** Błażej Misiak, Dorota Frydecka, Łukasz Łaczmański, Ryszard Ślęzak, Andrzej Kiejna

**Affiliations:** 1 Department of Psychiatry, Wroclaw Medical University, 10 Pasteur Street, 50-367 Wroclaw, Poland; 2 Department of Genetics, Wroclaw Medical University, 1 Marcinkowski Street, 50-368 Wroclaw, Poland; 3 Department of Endocrinology and Diabetology, Wroclaw Medical University, 4 Pasteur Street, 50-367 Wroclaw, Poland

**Keywords:** One-carbon metabolism, Homocysteine, First-episode schizophrenia, Second-generation antipsychotics, Olanzapine, Risperidone

## Abstract

**Purpose:**

Alterations in one-carbon metabolism (OCM) have been repeatedly reported in schizophrenia. However, there is a scarcity of studies addressing the effects of antipsychotics on selected OCM markers in schizophrenia and provided results are inconsistent.

**Methods:**

We recruited 39 first-episode schizophrenia (FES) patients and determined serum profile of total homocysteine (tHcy), folate, vitamin B12, lipoproteins and glucose at baseline and after 12 weeks of treatment with second-generation antipsychotics (SGA) including olanzapine and risperidone in monotherapy.

**Results:**

After 12 weeks of treatment, all patients had significantly higher body mass index (BMI), serum levels of total cholesterol (TC), low-density lipoproteins (LDL), triglycerides (TG) and tHcy together with significantly lower levels of folate and vitamin B12. The analysis of differences between SGA revealed the same biochemical alterations in patients treated with olanzapine as in the whole group, while those receiving risperidone had no statistically significant changes in serum folate, vitamin B12 and TG. There was a significantly higher increase in BMI and TC in patients treated with olanzapine in comparison with those treated with risperidone. Patients receiving olanzapine had a higher decrease in vitamin B12 than those assigned to the treatment with risperidone. Changes in folate, vitamin B12, tHcy and TC levels were significant only in males, even after Bonferroni correction. Multiple regression analysis revealed that changes in tHcy levels are associated with gender and baseline metabolic parameters (BMI, glucose, TC, LDL and HDL) but not with selected SGA.

**Conclusions:**

These results indicate that SGA may influence OCM, especially in first-episode schizophrenia (FES) males.

**Electronic supplementary material:**

The online version of this article (doi:10.1007/s00228-014-1762-2) contains supplementary material, which is available to authorized users.

## Introduction

Life expectancy in schizophrenia patients is about 20 years shorter in comparison with the general population [1]. This mortality gap has been widely attributed to high prevalence rates of medical comorbidities, mainly the metabolic syndrome (MetS), which is a clustering of conditions that increase cardiovascular risk including visceral obesity, impaired glucose metabolism, lipid profile disturbances and hypertension. There are studies indicating significantly higher prevalence of MetS among schizophrenia patients in comparison with the general population [2]. Although many factors are involved in the etiology of MetS, antipsychotic treatment serves as the major contributor of MetS development in schizophrenia [3]. Moreover, accumulating evidence indicates that even drug-naïve first-episode schizophrenia (FES) patients show signs of subclinical metabolic deregulation [4–8].

One-carbon metabolism (OCM) alterations including elevated plasma/serum homocysteine (Hcy) level together with decreased folate and vitamin B12 levels are frequently reported among FES patients [4, 8–11]. Two previous meta-analyses including studies on various subgroups of patients indicated that increased Hcy level is associated with higher schizophrenia risk [12, 13]. Several mechanisms may underlie causative links between OCM disturbances and schizophrenia. However, it seems that the interactions between Hcy and NMDA receptors seem to be the most relevant to schizophrenia pathophysiology [14]. Furthermore, OCM provides methyl groups for DNA methylation influencing gene expression patterns. Aberrant DNA methylation of genes involved in neurotransmission and brain development has been widely implicated in schizophrenia pathophysiology [15–17]. Moreover, methyl group transfer in transmethylation reactions of the OCM is required for the synthesis of catecholamines in the catechol-*O*-methyltransferase (COMT) reaction.

The recent study by Geller et al. [18] indicated that OCM deregulation in terms of elevated Hcy levels occurs also in siblings of schizophrenia patients. In addition, it has been found that schizophrenia patients with positive family history of schizophrenia are characterized by significantly higher Hcy and lower vitamin B12 levels in comparison to those without first-degree relatives with schizophrenia [4]. These findings suggest the role of genetic factors in OCM alterations in schizophrenia and are in line with the plethora of studies showing the association between two functional polymorphisms (C677T and A1298C) in the methylenetetrahydrofolate reductase (*MTHFR*) gene and schizophrenia risk [12, 13]. Notably, MTHFR converts 5,10-methylenetetrahydrofolate to 5-methyltetrahydrofolate. This reaction is involved in the re-methylation of Hcy to methionine, a reaction of great importance for tHcy levels in blood. It has been found that polymorphisms in the *MTHFR* gene may predict the development of MetS after the initiation of antipsychotic treatment [19–22]. There is also one study showing this relationship in drug-naïve FES patients [23].

Although the *MTHFR* gene polymorphisms have been repeatedly studied with respect to the development of MetS in response to antipsychotic treatment [21, 22, 20, 24], the influence of antipsychotics on OCM was investigated only in one observational study on FES patients [11]. The authors followed 22 drug-naïve FES patients after they started their initial antipsychotic treatment, showing no significant changes in Hcy level after 6 months of observation. Notably, this study was not sizable and did not include other measures of OCM activity such as vitamin B12 and folate. To provide a more comprehensive insight into OCM, we performed a more detailed observational study including the measurement of serum Hcy, folate, vitamin B12, lipoproteins and glucose level after the initiation of treatment with second-generation antipsychotics (SGA) in 39 FES patients.

## Material and methods

### Subjects

The total sample consisted of 39 FES patients (23 males with a mean age of 23.78 ± 3.87 years and 16 females with a mean age of 29.31 ± 5.36 years). Schizophrenia was diagnosed according to DSM-IV and ICD-10 criteria and confirmed using the Operational Criteria for Psychotic Illness (OPCRIT) checklist [25]. Additionally, current psychopathological manifestation was assessed using the positive and negative syndrome scale (PANSS) [26]. Assessment of cigarette smoking was performed using the pack-year index and the Fagerström questionnaire [27]. There were following exclusion criteria: mental retardation and/or general brain disorder, supplementation of folic acid or B vitamins, positive urine screening for illicit drugs (cannabis, amphetamine, opiates and ecstasy), drug and/or alcohol use disorder during 1 year prior to the onset of psychotic symptoms, severe somatic comorbidities and the use of medications altering OCM parameters (e.g. methotrexate, anticonvulsants, theophylline, proton-pump inhibitors, statins, fibrates, anti-hypertensive or anti-diabetic drugs). The study was approved by the local Ethics Committee, and all participants gave an informed consent for participation in the study. In addition, the study was performed in accordance with ethical standards laid down in the 1964 Declaration of Helsinki and its later amendments.

Patients were assigned to the treatment with oral antipsychotics monotherapy: olanzapine (24 patients, maximum dose 20 mg/day) and risperidone (15 patients, maximum dose 8 mg/day) and were followed up after 12 weeks. There were 10 drug-naïve patients on the day of recruitment, while other patients had a low dose of the above-mentioned antipsychotics (olanzapine up to 10 mg/day and risperidone up to 3 mg/day). Mean treatment duration on the day of recruitment was 5.54 ± 4.37 days, and chlorpromazine (CPZ) equivalent was 138.09 ± 117.81 mg/day. Agitation and hostility were treated using haloperidol and benzodiazepines.

Anthropometric and metabolic parameters including weight, height, body mass index (BMI), serum levels of low-density lipoproteins (LDL), high-density lipoproteins (HDL), total cholesterol (TC), triglycerides (TG), glucose, total Hcy (tHcy), folate and vitamin B12 were assessed on the day of recruitment and after 12 weeks of antipsychotic treatment as described in our previous article [4]. Data on serum creatinine levels were obtained from hospital case notes. Serum creatinine level was measured within 3 days from the day of admission to the hospital using the Jaffe reaction assay in the Cobas 6000 analyzer (Roche, Switzerland). Blood samples were obtained between 7.30 and 8.30 a.m. after at least 10-h overnight fasting. All samples were centrifuged about 10 a.m. The same time interval between blood sampling and centrifugation was maintained for all the patients at baseline and follow-up measurements.

## Statistics

A paired sample *t* test was used to compare differences in TC before and after antipsychotic treatment, which was a normally distributed variable. Other parameters before and after antipsychotic treatment were not normally distributed and thus were compared using a Wilcoxon test. Baseline characteristics of FES patients assigned to the treatment with olanzapine and risperidone were compared using Mann–Whitney *U* test (biochemical parameters, BMI and PANSS scores), Fisher test (education) and *χ* ^2^ test (gender distribution). Similarly, Mann–Whitney *U* test was used to compare percent of changes in studied parameters between patients treated with olanzapine and those receiving risperidone. Correlations between baseline CPZ and biochemical or clinical parameters, as well as between serum levels of tHcy, folate or vitamin B12 and the scores of PANSS subscales were assessed using the Spearman rank correlation coefficient. Multiple regression model was performed to indicate the predictors of a relative change of tHcy level. The model included age, gender, type of antipsychotic treatment (olanzapine or risperidone), pack-year index, Fagerström test score, baseline metabolic parameters (BMI, creatinine, glucose, TC, TG, HDL, LDL, tHcy, folate and vitamin B12) and their relative changes, baseline treatment duration and daily chlorpromazine equivalent as well as baseline score of negative symptoms as measured by PANSS and a relative change of it. Differences were considered as statistically significant if the *p* value was less than 0.05. Due to multiple comparisons, Bonferroni correction was applied to the level of significance. Statistical analyses were performed using the STATISTICA 10 software.

## Results

There were no significant differences in the baseline socio-demographic, clinical, biochemical and anthropometric measurements between patients assigned to treatment with olanzapine and risperidone; however, CPZ was significantly higher in patients treated with olanzapine in comparison with those treated with risperidone on the day of recruitment (*p* < 0.001), and this difference remained significant after Bonferroni correction (*p* < 0.0028) (Table 1). Baseline CPZ was positively correlated with the increase in TC in the whole group (*r* = 0.426, *p* = 0.007), as well as separately in patients treated with olanzapine (*r* = 0.255, *p* = 0.027) and risperidone (*r* = 0.468, *p* = 0.048). There was no significant correlation between CPZ and changes in other clinical or metabolic parameters (data not shown). Similarly, the indices of nicotine dependence (the Fagerström test score and the pack-year index) did not correlate with baseline and follow-up biochemical parameters with exception of serum LDL level that positively correlated with the pack-year index at the follow-up time point (*r* = 0.340, *p* = 0.027).

**Table 1:** The comparison of baseline characteristics of patients assigned to the treatment with olanzapine and risperidone

	The whole group (*N* = 39)	Olanzapine (*N* = 24)	Risperidone (*N* = 15)	*p* value^a^
Age	26.0 ± 5.3	25.2 ± 5.3	27.3 ± 4.7	0.141
Gender (M/F)	39	16/8	7/8	0.217
Education (higher/other than higher)	6/33	4/20	2/13	0.779
BMI (kg/m^2^)	23.5 ± 4.1	23.4 ± 4.3	23.6 ± 3.7	0.751
Glucose (mg/dl)	84.1 ± 7.5	83.2 ± 7.4	85.5 ± 7.7	0.378
LDL (mg/dl)	95.3 ± 28.0	97.0 ± 30.5	92.7 ± 24.3	0.919
HDL (mg/dl)	53.3 ± 15.0	52.6 ± 15.7	54.3 ± 14.3	0.654
TC (mg/dl)	175.8 ± 35.6	177.2 ± 37.0	173.5 ± 34.2	0.686
TG (mg/dl)	121.5 ± 83.1	132.7 ± 88.3	103.6 ± 73.3	0.161
tHcy (μmol/l)	11.9 ± 4.2	12.0± 3.9	11.7 ± 4.8	0.623
Folate (ng/ml)	6.9 ± 2.6	6.8 ± 2.6	7.0 ± 2.8	0.919
Vitamin B12 (pg/ml)	405.2 ± 201.4	392.1 ± 182.2	426.1 ± 234.2	0.919
Creatinine level (mg/dl)	0.8 ± 0.1	0.8 ± 0.1	0.7 ± 0.1	0.057
Fagerstrom score	1.6 ± 2.7	1.3 ± 2.4	2.0 ± 3.2	0.634
Pack-year index	1.8 ± 3.2	1.4 ± 2.9	2.3 ± 3.6	0.573
Treatment duration (days)	5.1 ± 4.0	5.9 ± 3.7	4.0 ± 4.4	0.204
CPZ (mg/day)	135.9 ± 121.4	185.4 ± 124.6	56.7 ± 59.4	<*0.001* ^†^
PANSS—positive symptoms score	24.1 ± 4.9	25.3 ± 5.3	22.1 ± 3.4	0.061
PANSS—negative symptoms score	19.0 ± 7.2	18.5 ± 7.1	19.7 ± 7.4	0.707
PANSS—general psychopathology score	42.8 ± 7.4	44.0 ± 7.7	40.9 ± 6.7	0.333

Data expressed as mean ± SD with exception of gender distribution (number of cases)

*BMI* body mass index, *CPZ* chlorpromazine equivalent, *HDL* high-density lipoproteins, *LDL* low-density lipoproteins, *PANSS* the Positive and Negative Syndrome Scale, *TC* total cholesterol, *tHcy* total homocysteine

^†^ *p* < 0.0028, *p* value significant after application of Bonferroni correction (*p* < 0.0028); significant difference (*p* < 0.05) was marked in italics

^a^ *p* value refers to the comparison of mean ranks of patients assigned to the treatment with olanzapine and those receiving risperidone using Mann–Whitney *U* test with exception of gender and education distribution assessed using *χ* ^2^ test and Fisher’s test

Clinical characteristics, as well as anthropometric and metabolic indices in the course of antipsychotic treatment, are shown in Table 2. Antipsychotic treatment with olanzapine and risperidone resulted in a significant increase in BMI, TC, LDL, TG and tHcy (*p* < 0.001). There was no significant change in serum glucose level and HDL (*p* > 0.05). Serum levels of vitamin B12 and folate were significantly lower after 12 weeks of antipsychotic treatment (*p* = 0.013 and *p* < 0.001, respectively). Simultaneously, antipsychotic treatment was associated with significant improvement of psychopathology as measured by PANSS (*p* < 0.001). All these changes, with exception of the decrease in vitamin B12 level, remained significant after Bonferroni correction (*p* < 0.0041). Changes in tHcy levels were significant in all tertiles calculated from baseline folate and vitamin B12 levels (*p* < 0.05).

**Table 2:** Clinical characteristics, anthropometric and metabolic measures in the course of antipsychotic treatment

	At baseline	After 12 weeks	*p* value^a^
BMI (kg/m^2^)	23.5 ± 4.1 (23.2)	24.4 ± 4.5 (23.9)	<*0.001* ^†^
Glucose (mg/dl)	84.1 ± 7.5 (85.0)	85.1 ± 5.5 (85.8)	0.118
LDL (mg/dl)	95.3 ± 28.0 (94.3)	116.9 ± 36.8 (111.8)	<*0.001* ^†^
HDL (mg/dl)	53.3 ± 15.0 (53.1)	56.00 ± 18.3 (53.9)	0.515
TC (mg/dl)	175.8 ± 35.6 (175.0)	192.1 ± 37.1 (187.0)	<*0.001* ^†^
TG (mg/dl)	121.5 ± 83.1 (95.0)	139.0 ± 85.9 (119.0)	<*0.001* ^†^
tHcy (μmol/l)	11.9 ± 4.2 (11.4)	12.9 ± 4.3 (11.9)	<*0.001* ^†^
Folate (ng/ml)	6.9 ± 2.6 (6.2)	5.9 ± 2.2 (6.1)	<*0.001* ^†^
Vitamin B12 (pg/ml)	405.2 ± 201.4 (353.1)	376.9 ± 159.5 (326.4)	*0.006*
PANSS—positive symptoms score	24.1 ± 4.9 (24.0)	8.0 ± 2.2 (7.0)	<*0.001* ^†^
PANSS—negative symptoms score	19.0 ± 7.2 (17.0)	14.8 ± 5.0 (13.0)	<*0.001* ^†^
PANSS—general psychopathology score	42.8 ± 7.4 (42.00)	19.8 ± 4.8 (18.0)	<*0.001* ^†^

Data expressed as mean ± SD (median)

*BMI* body mass index, *HDL* high-density lipoproteins, *LDL* low-density lipoproteins, *PANSS* the Positive and Negative Syndrome Scale, *TC* total cholesterol, *tHcy* total homocysteine

^†^ *p* < 0.0041, *p* value significant after application of Bonferroni correction; significant differences (*p* < 0.05) are marked in italics

^a^ *p* value calculated using a Wilcoxon test with exception of TC (*p* value calculated using a paired sample *t* test)

Treatment with olanzapine was associated with a significant increase in BMI (23.4 ± 4.3 vs. 24.5 ± 4.6 kg/m^2^, *p* < 0.001), TC (177.2 ± 37.0 vs. 195.6 ± 39.1 mg/dl, *p* < 0.001), LDL (97.0 ± 30.5 vs. 122.3 ± 36.1 mg/dl, *p* < 0.001), TG (132.7 ± 88.3 vs. 149.4 ± 87.3 mg/dl, *p* < 0.001) and tHcy (12.0 ± 3.9 vs. 13.0 ± 4.2 μmol/l, *p* = 0.003) (Supplementary Table 1). In addition, the treatment with olanzapine resulted in a significant decrease in serum levels of vitamin B12 (392.1 ± 182.2 vs. 357.3 ± 168.4 pg/ml, *p* = 0.001) and folate (6.8 ± 2.6 vs. 6.0 ± 2.3 ng/ml, *p* < 0.001). There was no significant change in the level of serum glucose (83.2 ± 7.4 vs. 84.2 ± 5.7 mg/dl, *p* = 0.393) and HDL (52.6 ± 15.7 vs. 53.3 ± 16.5 mg/dl, *p* = 0.063) during the treatment with olanzapine. Patients treated with olanzapine improved significantly in terms of the severity of positive symptoms (25.3 ± 5.3 vs. 7.8 ± 1.6), negative symptoms (18.5 ± 7.1 vs. 14.5 ± 4.7) and general psychopathology (44.0 ± 7.7 vs. 19.1 ± 3.8) as assessed using PANSS (*p* < 0.001).

The change in metabolic parameters was less pronounced in the group treated with risperidone (Supplementary Table 1). Pharmacotherapy with risperidone was associated with a significant increase in BMI (23.6 ± 3.7 vs. 24.4 ± 3.9 kg/m^2^, *p* = 0.001), LDL (92.7 ± 24.3 vs. 114.8 ± 39.3 mg/dl, *p* = 0.001), TC (173.5 ± 34.2 vs. 185.9 ± 34.1 mg/dl, *p* = 0.003) and tHcy (11.7 ± 4.8 vs. 12.5 ± 4.5 μmol/). There were no significant changes (*p* > 0.05) in the levels of serum glucose (85.5 ± 7.7 vs. 87.2 ± 4.7 mg/dl), TG (103.6 ± 73.3 vs. 115.8 ± 80.9), HDL (54.3 ± 14.3 vs. 60.3 ± 20.7 mg/dl), folate (7.0 ± 2.8 vs. 6.3 ± 2.1 ng/ml) and vitamin B12 (426.1 ± 234.2 vs. 385.8 ± 149.3 pg/ml). Similar to olanzapine, the treatment with risperidone resulted in a significant improvement (*p* < 0.001) with respect to the severity of positive symptoms (22.1 ± 3.4 vs. 8.5 ± 2.9), negative symptoms (19.7 ± 7.4 vs. 14.9 ± 5.5) and general psychopathology (40.9 ± 6.7 vs. 20.7 ± 6.2) evaluated using PANSS. Changes in BMI, LDL, TC, TG, tHcy, folate, vitamin B12 and all PANSS subscales in patients receiving olanzapine, as well as changes in BMI, LDL, TC and all PANSS subscales in those treated with risperidone remained significant after Bonferroni correction (*p* < 0.004).

Patients receiving olanzapine, in comparison with those treated with risperidone, had significantly higher percent of changes in BMI (4.4 ± 1.5 vs. 3.2 ± 1.3 %, *p* = 0.020) and TC (11.0 ± 5.7 vs. 7.7 ± 7.9 %, *p* = 0.028). In addition, FES patients assigned to the treatment with olanzapine had significantly higher decrease in vitamin B12 level than those receiving risperidone (−5.0 ± 5.1 vs. −1.6 ± 2.1 %, *p* = 0.019). Significantly higher improvement of positive symptoms assessed using PANSS was observed in patients treated with olanzapine than those receiving risperidone (−68.5 ± 6.8 vs. −61.2 ± 12.6 %, *p* = 0.017). There was no significant difference in the change in serum glucose, LDL, HDL, TG, folate, tHcy and the severity of negative symptoms or general psychopathology assessed with PANSS (*p* > 0.05). None of these differences remained significant after Bonferroni correction (*p* > 0.0041).

Baseline tHcy levels exhibited a weak positive correlation with severity of negative symptoms at baseline (*r* = 0.572, *p* < 0.001) and at follow-up (*r* = 0.539, p < 0.001). Similarly, follow-up tHcy level positively correlated with the severity of negative symptoms after treatment (*r* = 0.554, *p* < 0.001).

Changes in metabolic parameters with respect to gender are shown in Supplementary Table 2. In males, antipsychotic treatment was associated with a significant increase in BMI (24.5 ± 3.9 vs. 25.4 ± 4.1 kg/m^2^, *p* < 0.001), LDL (92.8 ± 26.8 vs. 116.1 ± 36.9 mg/dl, *p* < 0.001), TC (174.6 ± 40.2 vs. 192.8 ± 41.4 mg/dl, *p* < 0.001), TG (125.3 ± 102.5 mg/dl, *p* < 0.001) and tHcy (12.2 ± 3.6 vs. 13.0 ± 3.9 μmol/l, *p* = 0.002) as well as a significant decrease in serum levels of folate (6.3 ± 2.4 vs. 5.3 ± 2.2 ng/ml, *p* < 0.001) and vitamin B12 (421.5 ± 192.5 vs. 402.3 ± 176.5 pg/ml, *p* = 0.002). In addition, treatment with olanzapine and risperidone was associated with a significant improvement of positive (24.3 ± 5.7 vs. 8.2 ± 2.6, *p* < 0.001), negative (19.0 ± 7.1 vs. 15.1 ± 4.7) and general psychopathology (42.1 ± 7.1 vs. 20.2 ± 5.8, *p* < 0.001) symptoms as measured by PANSS. These changes were significant after Bonferroni correction (*p* < 0.003). In females, antipsychotic treatment was related to a significant increase in BMI (22.1 ± 4.0 vs. 23.1 ± 4.2 kg/m^2^, *p* < 0.001), TC (177.4 ± 28.8 vs. 191.2 ± 31.1 mg/dl, *p* < 0.001), LDL (99.0 ± 30.2 vs. 118.0 ± 38.1 mg/dl, *p* < 0.001), TG (116.1 ± 44.8 vs. 129.6 ± 52.8 mg/dl, *p* = 0.031) and tHcy (11.4 ± 5.1 vs. 12.8 ± 4.9 μmol/l, *p* = 0.019). As similar to males, antipsychotic treatment improved significantly scores of positive (23.7 ± 3.4 vs. 7.8 ± 1.2, *p* < 0.001), negative (18.9 ± 7.5 vs. 14.4 ± 5.6, p < 0.001) and general psychopathology symptoms (43.8 ± 7.9 vs. 19.2 ± 3.1, *p* < 0.001) as measured by PANSS. However, changes in TG and tHcy levels were not significant after Bonferroni correction (*p* > 0.003).

Multiple regression analysis revealed that a relative change of tHcy was associated with gender (*B* = 0.62, *t* = 3.17, *p* = 0.009) and baseline metabolic parameters including BMI (*B* = 0.57, *t* = 3.25, *p* = 0.008), glucose (*B* = −0.59, t = −2.35, *p* = 0.038), TC (*B* = −2.40, *t* = −3.71, *p* = 0.003), LDL (*B* = 2.21, *t* = 3.91, *p* = 0.002) and HDL (*B* = 1.69, *t* = 4.34, *p* = 0.001) levels (Supplementary Table 3).

## Discussion

In this study, we found that the treatment with SGA may worsen metabolic profile in terms of decreasing vitamin B12 and folate levels and increasing Hcy levels. There were moderate differences between olanzapine and risperidone with respect to OCM alterations, which did not remain significant after correction for multiple testing. Similarly, multiple regression analysis revealed that olanzapine and risperidone have similar influence on tHcy changes. Both antipsychotics may lead to the increase in tHcy levels. Furthermore, we found that only treatment with olanzapine was associated with decreased folate and vitamin B12 levels. However, the comparison of these antipsychotics revealed a greater decrease in vitamin B12 levels during olanzapine treatment. There are studies that are in agreement with our results. For instance, Eren et al. [28] found that higher doses of typical antipsychotics (CPZ >400 mg/day) are associated with lower folate levels. Furthermore, it has been found that plasma *N*-desmethyl-olanzapine level positively correlates with Hcy level in schizophrenia patients treated with olanzapine [29].

It is now well-established that the treatment with SGA contributes to metabolic deregulation in terms of increased food intake, alterations in lipid profile and glucose metabolism [3]. There are also studies showing that the presence of MetS is associated with higher Hcy levels in schizophrenia patients as well as in the general population [30, 31]. However, it remains controversial as to whether MetS related to the use of some SGA increases mortality rate in schizophrenia. For instance, the recent 11-year longitudinal study from Finland revealed that clozapine, an antipsychotic drug known to induce adverse metabolic changes, was associated with a substantially lower mortality [32]. In this regard, it should be noted that we did not assess OCM alterations with respect to clinical outcome. Differences between olanzapine and risperidone with respect to OCM alterations correspond with previous studies showing higher propensity of olanzapine to induce weight gain and adverse metabolic effects in comparison with risperidone [33]. In agreement with previous studies on FES patients [34–36], especially with respect to weight gain, we found a higher increase in BMI and TC in patients receiving olanzapine in comparison with those treated with risperidone. However, the exact mechanism of antipsychotics’ action with respect to OCM remains unknown. Two principal mechanisms may explain higher tHcy and lower folate and B12 levels in response to treatment with antipsychotics—either decreased food vitamin intake or increased demands. Given that patients’ diet was similar in the course of the study as they were treated in the same hospital and changes in tHcy were similar in all tertiles of baseline folate and vitamin B12 levels, it is most likely that increased demands underlie presented findings. This hypothesis is in agreement with the study by Melka et al. [37], who investigated genome-wide DNA methylation in the hippocampus and cerebellum of rat animal models showing that olanzapine causes increase in DNA methylation of several genes, including those involved in metabolic homeostasis. In addition, MetS or its single components are associated with Hcy level and thus it is likely that alterations in OCM are secondary to metabolic changes induced by SGA.

It should be noted that some previous studies are not in agreement with our findings. There is only one previous study addressing changes in Hcy levels in response to the treatment with SGA. However, the authors did not find a significant change in Hcy levels in the course of a 6-month antipsychotic treatment of 22 FES patients [11]. Another study on 40 schizophrenia patients with a 6-week follow-up did not also reveal any significant change in serum Hcy [38]. In contrast to these results, Petronijevic et al. [39] found that schizophrenia patients have significantly higher Hcy levels in acute relapse than during remission phase. There is also one study showing no significant difference in Hcy levels between patients receiving clozapine in monotherapy in comparison with healthy controls [40]. These discordant results might be due to the differences in sample sizes, the lack of assessment of dietary intake, differences in the severity of psychopathological symptoms after treatment across studies as well as the lack of *MTHFR* genotyping. Indeed, it has been found that the *MTHFR* gene polymorphisms influence Hcy levels [41] and might predict the development of MetS components in schizophrenia patients [19–23]. This is even more important, when small patient samples are assessed, in which some genotypes might be over- or underrepresented.

Several studies have provided evidence that Hcy levels are positively correlated with the severity of negative symptoms [42, 4, 39, 43, 44]. Additionally, it has been demonstrated that polymorphisms in the *MTHFR* gene are associated with negative symptoms severity [45, 46]. The exact mechanism of the correlation between Hcy levels and the severity of negative symptoms remains unknown. However, it cannot be excluded that this association is the consequence of Hcy interactions within NMDA receptors [14]. In this study, we found that higher baseline tHcy level is associated with higher severity of negative symptoms after 12 weeks of treatment with SGA. At this point, it should be noted that while current antipsychotics exert high efficacy with respect to the treatment of positive symptoms, their effects on negative symptoms or cognitive deficits leave much to be desired [47]. It is also increasingly speculated that antipsychotic treatment, most likely due to unfavourable metabolic changes and subsequent cardiovascular complications, may indirectly contribute to progressive brain changes [48]. In addition, it should be noted that folate and vitamin B12 supplementation strategy has been proved to be effective as the add-on treatment of negative symptoms in schizophrenia. This has been shown with respect to polymorphisms of genes involved in OCM including *MTHFR* [49] and *FOLH1*, which encodes folate hydrolase involved in folate absorption [50].

Finally, we found that only FES males might develop lower vitamin B12 and folate levels as well as higher tHcy levels in response to treatment with olanzapine or risperidone. These findings are in agreement with studies showing higher Hcy levels in schizophrenia patients [51, 52] and the general population [53, 54]. The exact mechanisms of gender differences with respect to Hcy metabolism remain unclear. However, there are studies showing that androgens may increase Hcy levels [55].

Our results should be interpreted with caution due to limitations that require further discussion. Firstly, it should be noted that our sample size was limited; however, to our knowledge, it is the largest study on FES patients investigating the effects of antipsychotics on OCM. Furthermore, it should be noted that the majority of included patients were not drug-naïve; however, they had a low dose of antipsychotics and treatment duration was relatively short. In our previous study [4], which partially included the patients assessed in this study, we found that there was no significant correlation between lipid profile parameters, BMI, serum glucose, tHcy, folate or vitamin B12 and treatment duration or CPZ on the day of recruitment. Another point is that treatment allocation was not random and patients assigned to risperidone and olanzapine differed significantly with regard to baseline CPZ equivalent with higher baseline dose in those assigned to olanzapine treatment. However, baseline CPZ correlated only with TC level. Furthermore, we did not measure methylmalonic acid level that would have allowed differentiation between impaired folate and vitamin B12 status as well as we did not genotype the *MTHFR* polymorphisms that may alter the levels of OCM markers. The lack of other OCM markers including e.g. vitamins B2 and B6 might also be perceived as the limitation. In addition, it should be noted that tHcy was measured in serum that may cause higher tHcy levels due to its active extracellular transport [56]. However, the same pre-analytical conditions were maintained for all patients at baseline and follow-up. Finally, we did not assess dietary intake throughout the study but it should be noted that all patients were treated in the same hospital and thus had similar diets.

The results of our study may motivate routine assessment of OCM alterations in the course of schizophrenia, especially in males, and support the results of previous studies indicating the efficacy of vitamin supplementation strategies with respect to the severity of negative symptoms. Reported between drug differences indicate that assessment of OCM in addition to lipid status in FES might be useful in selecting antipsychotic treatment strategy. However, due to above-mentioned limitations, replication of our results in a larger and more comprehensive material is required.

## Electronic supplementary material

Below is the link to the electronic supplementary material.

Supplementary Table 1 (DOCX 112 kb)

Supplementary Table 2 (DOCX 97.6 kb)

Supplementary Table 3 (DOCX 15.4 kb)
