# Supplementary material for: Effects of second-generation antipsychotics on selected markers of one-carbon metabolism and metabolic syndrome components in first-episode schizophrenia patients
Source: Eur J Clin Pharmacol. 2014 Oct 8;70(12):1433–41. doi: 10.1007/s00228-014-1762-2 (PMC4226930; doi:10.1007/s00228-014-1762-2)
Supplement: Supplementary file 1 — (DOCX 112 kb) [file 228_2014_1762_MOESM1_ESM.docx]

**Supplementary table 1.** The comparison of olanzapine and risperidone with respect to metabolic changes.

|  | **Olanzapine (N = 24)** | | | **Risperidone (N = 15)** | | | **Relative change^Ŧ^** | | |
| --- | --- | --- | --- | --- | --- | --- | --- | --- | --- |
|  | **At baseline** | **After 12 weeks** | **p*** | **At baseline** | **After 12 weeks** | **p*** | **Olanzapine** | **Risperidone** | **p**** |
| BMI (kg/m^2^) | 23.4 ± 4.3 (23.1) | 24.5 ± 4.6 (24.6) | **< 0.001**^†^ | 23.6 ± 3.7  (23.18) | 24.4 ± 3.9  (23.7) | **0.001**^†^ | 4.4 ± 1.5 (4.4) | 3.2 ± 1.3 (3.5) | **0.020** |
| Glucose (mg/dl) | 83.2 ± 7.4  (83.4) | 84.2 ± 5.7  (83.4) | 0.393 | 85.5 ± 7.7  (87.8) | 87.2 ± 4.7  (87.0) | 0.330 | 0.9 ± 5.3 (1.6) | 2.6 ± 9.5 (3.9) | 0.367 |
| LDL (mg/dl) | 97.0 ± 30.5 (93.5) | 122.3 ± 36.1  (111.3) | **< 0.001**^†^ | 92.7 ± 24.3 (94.3) | 114.8 ± 39.3  112.3 | **0.001**^†^ | 25.3 ± 31.8 (17.6) | 24.2 ± 26.7 (18.2) | 0.875 |
| HDL (mg/dl) | 52.6 ± 15.7 (52.60) | 53.30 ± 16.5 (52.9) | 0.063 | 54.3 ± 14.3  (53.8) | 60.3 ± 20.7 (54.7) | 0.729 | 1.5 ± 13.5 (0.2) | 2.8 ± 17.7 (-1.0) | 0.282 |
| TC (mg/dl) | 177.2 ± 37.0 (181.5) | 195.6 ± 39.1 (199.0) | **< 0.001**^†^ | 173.5 ± 34.2 (173.0) | 185.9 ± 34.1 (182.0) | **0.003**^†^ | 11.0 ± 5.7 (10.1) | 7.7 ± 7.9 (6.7) | **0.028** |
| TG (mg/dl) | 132.7 ± 88.3 (104.0) | 149.4 ± 87.3 (126.5) | **< 0.001**^†^ | 103.6 ± 73.3 (83.0) | 115.8 ± 80.9 (98.0) | 0.140 | 53.6 ± 87.3 (26.5) | 15.8 ± 80.9 (12.5) | 0.054 |
| tHcy (μmol/l) | 12.0 ± 3.9  (11.6) | 13.0 ± 4.2  (12.1) | **0.003**^†^ | 11.7 ± 4.8  (11.1) | 12.5 ± 4.5 (11.6) | **0.019** | 12.8 ± 20.8 (10.9) | 11.7 ± 24.6 (13.0) | 0.597 |
| Folate (ng/ml) | 6.8 ± 2.6  (6.3) | 6.0 ± 2.3  (6.1) | **< 0.001**^†^ | 7.0 ± 2.8  (5.9) | 6.3 ± 2.1  (5.3) | 0.245 | -15.8 ± 11.8 (-17.7) | -7.3 ± 21.6 (-9.7) | 0.097 |
| Vitamin B12 (pg/ml) | 392.1 ± 201.4 (349.2) | 357.3 ± 168.4  (322.5) | **0.001**^†^ | 426.1 ± 234.2  (354.4) | 385.8 ± 149.3  (335.0) | 0.683 | -5.0 ± 5.1 (-6.0) | -1.6 ± 2.1 (-2.6) | **0.019** |
| PANSS – positive symptoms score | 25.3 ± 5.3  (24.0) | 7.8 ± 1.6  (7.0) | **< 0.001**^†^ | 22.1 ± 3.4  (23.0) | 8.5 ± 2.9  (7.0) | **< 0.001**^†^ | -68.5 ± 6.8 (-68.6) | -61.2 ± 12.6 (-63.2) | **0.017** |
| PANSS – negative symptoms score | 18.5 ± 7.1  (15.0) | 14.5 ± 4.7  (13.0) | **< 0.001**^†^ | 19.7 ± 7.4  (18.0) | 14.9 ± 5.5  (13.0) | **< 0.001**^†^ | -18.0 ± 13.3 (-14.8) | -22.0 ± 14.0 (-27.8) | 0.337 |
| PANSS – general psychopathology score | 44.0 ± 7.7  (42.00) | 19.1 ± 3.8  (18.00) | **< 0.001**^†^ | 40.9 ± 6.7  (41.0) | 20.7 ± 6.2  (19.0) | **< 0.001**^†^ | -55.7 ± 8.1 (-56.1) | -48.3 ± 16.2 (-47.9) | 0.109 |

Data expressed as mean ± SD (median) * p-value calculated using Wilcoxon test with exception of TCh (p-value calculated using a paired sample t-test) ** p-value calculated using Mann-Whitney U test ^Ŧ^ (follow-up – baseline)/baseline × 100 Significant differences (p < 0.05) were marked in bold ^†^ p-value significant after application of Bonferroni correction (p < 0.0041) Abbreviations: BMI – body mass index, HDL – high density lipoproteins, LDL – low density lipoproteins, PANSS – Positive and Negative Syndrome Scale, TC – total cholesterol, tHcy – total homocysteine
