# Supplementary material for: Effects of second-generation antipsychotics on selected markers of one-carbon metabolism and metabolic syndrome components in first-episode schizophrenia patients
Source: Eur J Clin Pharmacol. 2014 Oct 8;70(12):1433–41. doi: 10.1007/s00228-014-1762-2 (PMC4226930; doi:10.1007/s00228-014-1762-2)
Supplement: Supplementary file 2 — (DOCX 97.6 kb) [file 228_2014_1762_MOESM2_ESM.docx]

**Supplementary table 2.** Changes in metabolic parameters with respect to gender.

|  | **Males (N = 23)** | | | **Females (N =16)** | | |
| --- | --- | --- | --- | --- | --- | --- |
|  | At baseline | After 3 months | p-value* | At baseline | After 3 months | p-value* |
| BMI (kg/m^2^) | 24.5 ± 3.9 (24.0) | 25.4 ± 4.1 (25.2) | **< 0.001**^†^ | 22.1 ± 4.0 (20.5) | 23.1 ± 4.2 (21.2) | **< 0.001**^†^ |
| Glucose (mg/dl) | 83.4 ± 7.3 (85.0) | 83.8 ± 5.5 (85.2) | 0.831 | 85.2 ± 7.9 (83.5) | 86.9 ± 5.1 (86.8) | 0.214 |
| LDL (mg/dl) | 92.8 ± 26.8 (89.0) | 116.1 ± 36.9 (110.7) | **< 0.001**^†^ | 99.0 ± 30.2 (98.5) | 118.0 ± 38.1 (112.9) | **< 0.001**^†^ |
| HDL (mg/dl) | 51.9 ± 17.8 (47.2) | 55.9 ± 21.9 (52.0) | 0.475 | 55.3 ± 9.9 (55.0) | 56.2 ± 11.9 (55.7) | 0.897 |
| TC (mg/dl) | 174.6 ± 40.2 (173.0) | 192.8 ± 41.4 (186.0) | **< 0.001**^†^ | 177.4 ± 28.8 (180.0) | 191.2 ± 31.1 (194.0) | **< 0.001**^†^ |
| TG (mg/dl) | 125.3 ± 102.5 (87.0) | 145.6 ± 103.6 (119.0) | **< 0.001**^†^ | 116.1 ± 44.8 (113.5) | 129.6 ± 52.8 (142.5) | **0.031** |
| tHcy (μmol/l) | 12.2 ± 3.6 (11.9) | 13.0 ± 3.9 (12.4) | **0.002**^†^ | 11.4 ± 5.1 (10.8) | 12.8 ± 4.9 (11.3) | **0.019** |
| Folate (ng/ml) | 6.3 ± 2.4 (6.0) | 5.3 ± 2.2 (4.7) | **< 0.001**^†^ | 7.6 ± 2.8 (6.9) | 6.8 ± 2.0 (6.7) | 0.071 |
| Vitamin B12 (pg/ml) | 421.5 ± 192.5 (354.4) | 402.3 ± 176.5 (331.5) | **0.002**^†^ | 381.8 ± 217.8 (315.6) | 340.4 ± 127.8 (317.7) | 0.214 |
| PANSS – positive symptoms score | 24.3 ± 5.7 (24.0) | 8.2 ± 2.6 (7.0) | **< 0.001**^†^ | 23.7 ± 3.4 (23.5) | 7.8 ± 1.2 (7.0) | **< 0.001**^†^ |
| PANSS – negative symptoms score | 19.0 ± 7.1 (18.0) | 15.1 ± 4.7 (13.0) | **< 0.001**^†^ | 18.9 ± 7.5 (15.5) | 14.4 ± 5.6 (12.5) | **0.001**^†^ |
| PAN SS – general psychopathology score | 42.1 ± 7.1 (42.0) | 20.2 ± 5.8 (18.0) | **< 0.001**^†^ | 43.8 ± 7.9 (43.0) | 19.2 ± 3.1 (18.0) | **< 0.001**^†^ |

Data expressed as mean ± SD (median) *p-value calculated using Wilcoxon test Significant differences (p < 0.05) were marked in bold ^†^ significant differences after Bonferroni correction (p < 0.003) Abbreviations: BMI – body mass index, HDL – high density lipoproteins, LDL – low density lipoproteins, PANSS – the Positive and Negative Syndrome Scale, TC – total cholesterol, tHcy – total homocysteine
