# Supplementary material for: Effects of second-generation antipsychotics on selected markers of one-carbon metabolism and metabolic syndrome components in first-episode schizophrenia patients
Source: Eur J Clin Pharmacol. 2014 Oct 8;70(12):1433–41. doi: 10.1007/s00228-014-1762-2 (PMC4226930; doi:10.1007/s00228-014-1762-2)
Supplement: Supplementary file 3 — (DOCX 15.4 kb) [file 228_2014_1762_MOESM3_ESM.docx]

**Supplementary table 3.** Results of multivariable regression analysis of variables influencing relative change of total homocysteine level.

|  | **B** | **t** | **p** |
| --- | --- | --- | --- |
| Age (years) | -0.37 | -1.74 | 0.108 |
| Gender | 0.62 | 3.17 | **0.009** |
| T1 - BMI (kg/m^2^) | 0.57 | 3.25 | **0.008** |
| Pack-year index | -0.25 | -0.42 | 0.678 |
| Fagerström test | 0.20 | 0.39 | 0.701 |
| T1 - glucose (mg/dl) | -0.59 | -2.35 | **0.038** |
| T1 – TC (mg/dl) | -2.40 | -3.71 | **0.003** |
| T1 – LDL (mg/dl) | 2.21 | 3.91 | **0.002** |
| T1 – HDL (mg/dl) | 1.69 | 4.34 | **0.001** |
| T1 – TG (mg/dl) | 1.39 | 1.93 | 0.079 |
| T1 – tHcy (μmol/l) | -0.14 | -0.69 | 0.505 |
| T1 – folate (ng/ml) | -0.11 | -0.39 | 0.700 |
| T1 - vitamin B12 (pg/ml) | 0.17 | 0.62 | 0.549 |
| T1 - creatinine (mg/dl) | 0.28 | 1.91 | 0.081 |
| Treatment duration (days) | -0.03 | -0.17 | 0.866 |
| CPZ (mg/day) | -0.17 | -0.66 | 0.523 |
| T1 - negative symptoms score (PANSS) | -0.26 | -1.27 | 0.227 |
| Δ BMI (%) | -0.36 | -2.08 | 0.061 |
| Δ glucose (%) | -0.16 | -0.62 | 0.547 |
| Δ TC (%) | 0.27 | 1.54 | 0.151 |
| Δ LDL (%) | -0.20 | -0.94 | 0.366 |
| Δ HDL (%) | 0.26 | 1.21 | 0.250 |
| Δ TG (%) | -0.16 | -0.21 | 0.838 |
| Δ folate (%) | -0.11 | -0.59 | 0.566 |
| Δ vitamin B12 (%) | 0.36 | 1.91 | 0.083 |
| Δ negative symptoms score (PANSS) (%) | -0.02 | -0.07 | 0.939 |
| Treatment (olanzapine/risperidone) | -0.03 | -0.07 | 0.946 |

Significant differences (p < 0.05) were marked in bold Abbreviations: BMI – body mass index, CPZ – chlorpromazine equivalent, Δ – relative change, HDL – high density lipoproteins, LDL – low density lipoproteins, PANSS – Positive and Negative Syndrome Scale, T1 – baseline measurement, TC – total cholesterol, tHcy – total homocysteine
